# Bone Texture Fractal Dimension Analysis of Ultrasound-Treated Bone around Implant Site: A Double-Blind Clinical Trial

**DOI:** 10.1155/2018/2672659

**Published:** 2018-04-15

**Authors:** Elaf Akram Abdulhameed, Natheer Hashim Al-Rawi, Asmaa Tahseen Uthman, Ab Rani Samsudin

**Affiliations:** ^1^College of Dental Medicine, University of Sharjah, Sharjah, UAE; ^2^College of Dental Medicine, Gulf Medical University, Ajman, UAE

## Abstract

**Objectives:**

To evaluate the efficacy of bone texture fractal dimension (FD) analysis method in predicting implant stability from intraoral periapical radiographs using two implant protocols.

**Materials and Methods:**

A double-blind clinical trial was conducted on 22 subjects who needed dental implants. The participants were randomized into two groups, the control group with standard implant protocol treatment and the intervention group with added low-intensity power ultrasound treatment (LIPUS) besides the standard implant protocol. The FD values of bone density were carried out on the mesial and distal sides of the implant on digital intraoral radiographs using the box-counting method. Both resonance frequency (RF) and fractal dimension (FD) were assessed in three time intervals: after surgery and before and after loading.

**Results:**

FD on both the mesial and distal sides serve as very good-to-excellent tests with high validity (ROC area exceeding 0.8) in predicting high implant stability (ISQ ≥ 70). The mesial side measurements were consistently better than the distal side among the intervention groups. The optimum cutoff value for the FD-mesial side that predicts a highly stable implant (ISQ ≥ 70) is ≥1.505. At this optimum cutoff value, the mesial side FD is associated with a perfect sensitivity (100%) and fairly high specificity (86.5%).

**Conclusion:**

The FD analysis could be recommended as an adjunctive quantitative method in prediction of the implant stability with very high sensitivity and specificity. This trial is registered with ISRCTN72648040.

## 1. Introduction

Sufficient bone quality is a prerequisite for a successful dental implant. Proper assessment of the bone quality is a primary objective to determine the osseointegration status. Excessive marginal bone loss after implant or following prosthesis may be seen in the first year [1]. Continuous bone resorption affects function and aesthetic; therefore, there are several ways recommended to restore and regenerate the bone such as advocating bone-grafting procedures, usage of growth factors, low-level laser therapy, and therapeutic ultrasound. Low-intensity pulsed ultrasound (LIPUS) stimulation is a safe noninvasive treatment, and it can accelerate bone regeneration [2]. In dentistry, LIPUS has been found to promote periodontal bone defect healing [3], bone regeneration after oral surgery [4], and osseointegration of an endosseous dental implant [5].

Histological analysis was usually considered the gold-standard method to evaluate successful osseointegration. However, due to the invasiveness of this method and related ethical issues, various imaging modalities have been proposed like intraoral radiographs, panoramic imaging, computed tomography (CT), cone beam computed tomography (CBCT), and micro-CT [6]. Periapical radiographs are traditionally interpreted by measuring peri-implant marginal bone loss. This method has been found with limited diagnostic value for early detection of bone changes [7].

Bone texture analysis provides information about bone structures in a noninvasive manner [8, 9]. Fractal dimension analysis is one of the noninvasive, well-suited methods to analyze the bone texture on a plain radiograph [10]. This method is used to quantify the trabecular bone pattern and bone marrow interface using the box-counting logarithm [11, 12]. The fractal analysis is a statistical analysis of texture based on fractal geometry for describing complex structural patterns recognized and expressed as a ratio termed as the “fractal dimension” FD [13, 14]. The FD method has been used to quantify trabecular bone structures under different conditions, like endodontic treatment [15], periodontitis [16, 17], and implant stability [18, 19]. Jolley et al. [20] showed that the FD can reliably analyze the changes in the alveolar bone density by using the periapical radiographs.

There has been no report to determine the validity of intraoral periapical radiographs to predict the success rate of implant stability. Plethora of reports have tested the bone quality around the dental implants and compared it with the contralateral normal bone, but no study has been conducted to compare bone changes between two implant protocols [18, 19]. The aim of the present study was to evaluate the efficacy of the fractal dimension (FD) method in predicting stability around the dental implants treated with and without LIPUS from an intraoral periapical radiograph.

## 2. Materials and Methods

This randomized controlled clinical trial (RCT) was conducted on 22 patients attended to the University Dental Hospital Sharjah (UDHS) for dental implant therapy of single missing maxillary, first or second premolars. The age range was between 20 and 40 years (mean 33.13 ± 7.23 years). Patients with smoking habit or patients with any systemic diseases that might affect bone metabolism, or patients with parafunctional habits, and patients with active periodontal diseases or bad oral hygiene were excluded from the study. After explaining the study procedure, informed consent was obtained from all participants. The study participants were allocated randomly and equally divided into two groups; the control group (*n*=11) received the standard implant treatment, and the intervention group (*n*=11) which received low-intensity pulsed ultrasound (LIPUS) therapy in addition to the standard implant procedure.

**Figure 1:**
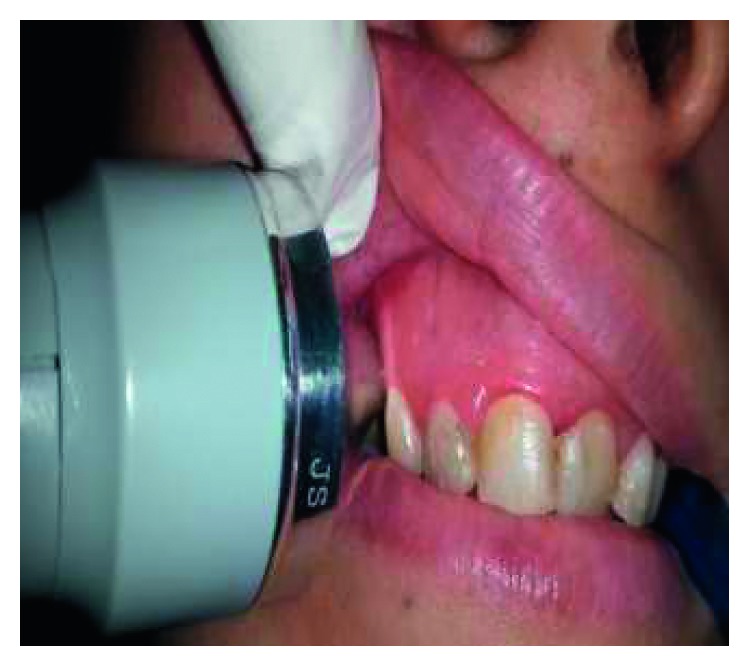
Ultrasound therapy delivered using probe on the buccal aspect of the implant site.

All patients underwent 2 stages of implant surgeries. Stage I implant surgery was performed with one SPI dental implant (Thommen Medical SPI Element MC Inicell) bone level type with a length of 9.5 mm, and a diameter of 4 mm was positioned in the maxillary edentulous premolar area in each patient. A stage II implant surgery was carried out after 2 months of implant placement where the dental implant was uncovered and impression was taken for crown placement. All patients received screw-retained porcelain fused to metal crown. The intervention group patients (*n*=11) were then subjected to LIPUS exposure two weeks following stage I implant surgery placement. The machine employed was Pulson ®330 Ultrasound Therapy unit (Gymna, Bilzen, Belgium) (Figure 1). According to Kerr et al. [21], the intensity of ultrasound therapy was set at 30 mW/cm^2^ with a frequency of 1.5 MHz and temporal average power of 20 mW. LIPUS was delivered intraorally on the buccal part of the implant site for a duration of 20 minutes twice a week started 2 weeks after dental implant placement and lasted for the subsequent 10 weeks.

The RF measurements and the FD measurements of bone density were done immediately after surgical dental implant placement and after three and six months, respectively.

For the RF analysis technique, the implant stability is estimated using an Osstell Mentor (Integration Diagnostics, Goteborg, Sweden) and Smart Pegs as described by Isoda et al. [22]. The Smart Pegs were mounted on the implants and tightened with a screw. The RF value was measured in four aspects (mesial, distal, buccal, and lingual) for each implant. The RF values were represented by a quantitative unit called the implant stability quotient (ISQ) on a scale from 1 to 100. High stability means >70 ISQ, between 60 and 69 is medium stability, and <60 ISQ is considered as low stability. The results were expressed in ISQ and averaged for each implant.

The fractal dimension analysis (FD) was made on digital intraoral radiographic images that were taken immediately after the placement of the dental implant and at 3 months and 6 months postoperatively using Image J software (https://imagej.nih.gov/ij/).

The region of interest (ROI) was set to 100 × 200 pixels (1.0 mm wide × 2.0 mm height) at the first macrothread around the mesial and distal aspects of each implant.

The region of interest (ROI) was cropped and was transferred to *Image J version 1.34s* by using the program menu. The saved images were processed using the White and Rudolph method [23]. ROI of duplicated image was blurred with a Gaussian filter (kernel size 35). The blurred image was then subtracted from the original image, and then, the resultant image was converted to binary by threshold at the gray value of 128 so that the segmented objects approximated the bony trabecular pattern. Finally, the image was skeletonized and was used for fractal analysis.

**Figure 2:**
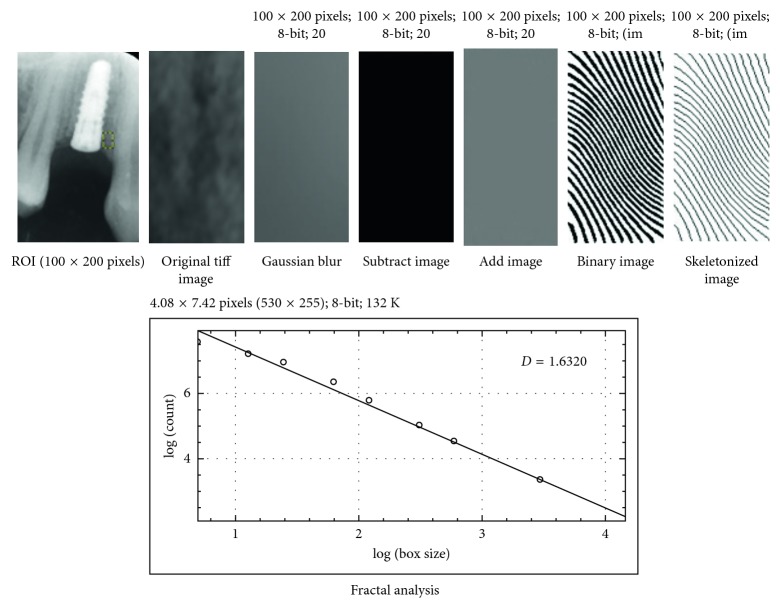
Image-processing procedure.

The fractal dimension of the skeletonized image was calculated using the box-counting function method mentioned by Demirbas et al. [24] The resulting numbers of the counted tiles (which refers to the trabecular bone) were plotted against the total number of the tiles in double logarithmic scale, and fractal dimension was calculated from the slope of the line fitted on the data points (Figure 2).

Data were expressed as mean and standard deviation (SD). Differences between groups were analyzed for significance using independent and paired *t*-test. A simple regression model was also used to predict the RF value based on measuring the FD. The statistical significance was defined as *P* < 0.05 using SPSS statistical package (SPSS, Version 24, Chicago, IL, USA).

## 3. Results

### 3.1. Reliability and Randomization

FD analyses were done by one investigator (EA), all measurements were repeated by the same investigator after 2 weeks, the intraexaminer reliability test was evaluated, and intraclass correlation coefficient test was measured. The intraclass correlation coefficient was measured first, and it was 0.965 indicating very high internal consistency. There were no obvious or statistically significant differences in age and gender composition between the two comparison groups. This qualifies as an evidence for adequate randomization process (Table 1).

The mean and the standard deviation of RF and FD values for the intervention and control groups are seen in (Table 2).

All the paired measurements of RF and FD values on all study participants (irrespective of their study groups and follow-up time) and all the three time intervals were used in a simple regression model. The model was used to predict the RF value based on measuring the FD.

### 3.2. Resonance Frequency Analysis

**Figure 3:**
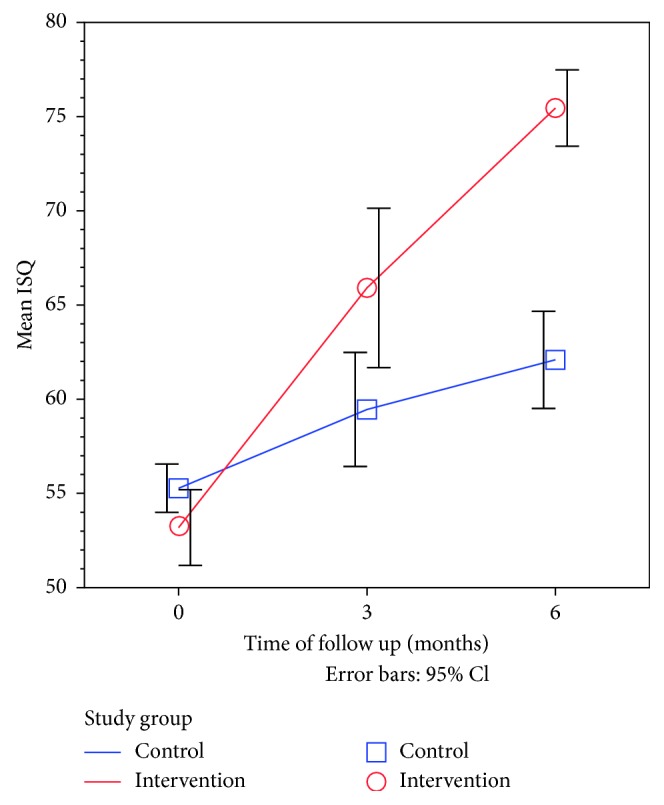
Line graph showing the time trend for mean RF values after surgery in the intervention group compared to the control group.

The mean RF measured immediately after surgery showed no important or statistically significant differences between the control and intervention groups (55.3% and 53.2%, resp.). This adds to the evidence for effective randomization process at the design stage (Table 3 and Figure 3).

The outcome of dental implant was assessed after three months and six months of surgery and compared to the immediate postoperative status. The mean change in RF values after these two time intervals was used to assess the magnitude of effect in the healing process in each study group. In addition, Cohen's *d* was used as a standardized measure of effect size to allow fair comparison of effect size between time intervals and between different types of measurements.

As shown in Table 3, after six months of follow-up, the RF values of the control group were significantly increased by a mean of 6.8 units in the control group. This effect was evaluated as a strong effect size (Cohen's *d* > 0.8). On the other hand, the RF values in the intervention group were significantly increased by a mean of 7.4 units after six months. This also translates to a very strong effect size (Cohen's *d* > 0.8). Moreover, the effect size after six months compared to immediate postoperative measurements was much stronger for the intervention group (Cohen's *d* = 7.4) compared to the control group (Cohen's *d* = 2.25). The extra benefit imposed on the intervention group was significantly higher than that on the control group.

### 3.3. Fractal Dimension Analysis

The mean FD measured immediately after surgery also showed no important or statistically significant differences between the control and intervention groups (1.338 and 1.319, resp.).

**Figure 4:**
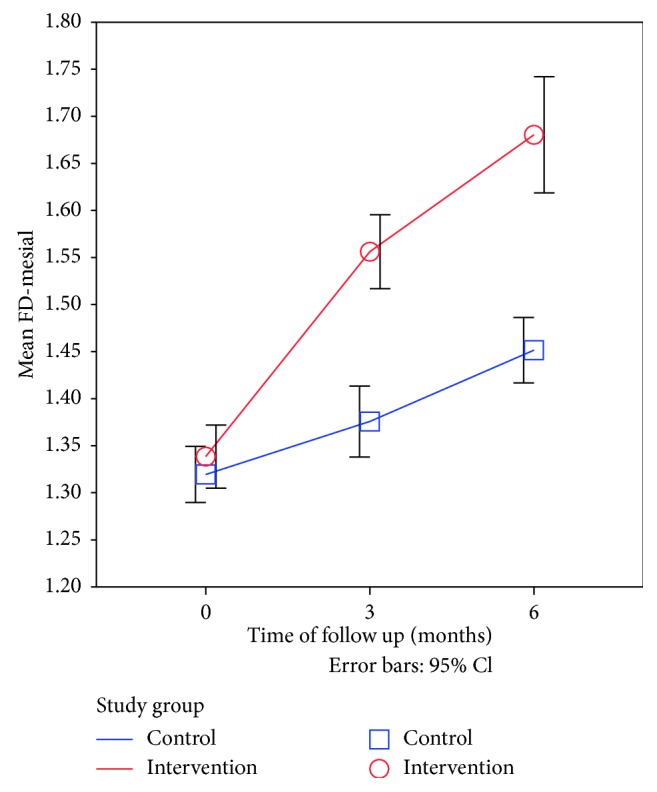
Line graph showing the time trend for mean FD-mesial side after surgery in the intervention group compared to the control group.

After six months of follow-up, the FD-mesial values were significantly increased by a mean of 0.132 units in the control group and 0.342 units in the intervention group which reflects a very strong effect size. Moreover, the effect size after six months compared to immediate postoperative measurements was much stronger for the intervention group (Cohen's *d* = 4.85) compared to the control group (Cohen's *d* = 2.64) (Table 4, Figure 4).

**Figure 5:**
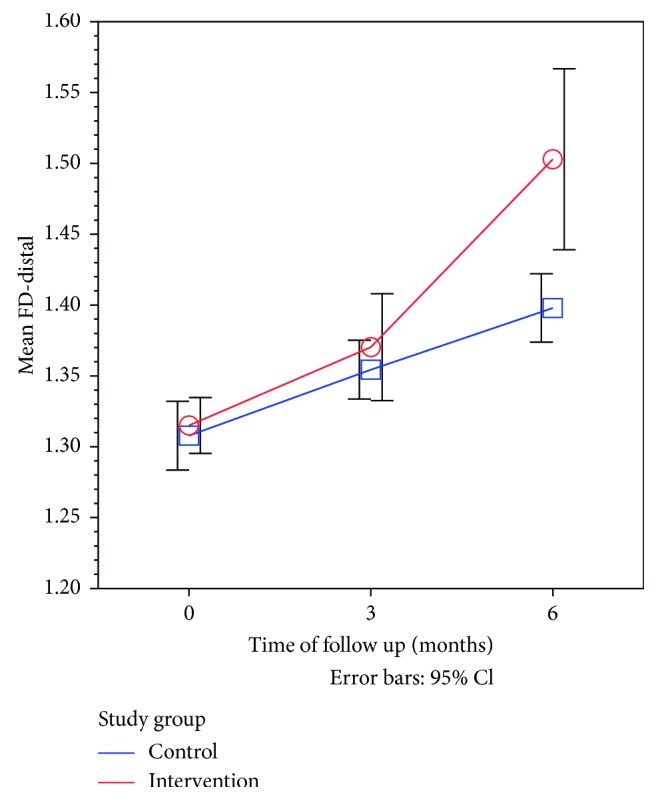
Line graph showing the time trend for mean FD-distal side after surgery in the intervention group compared to the control group.

Similarly, the FD-distal values were also increased on the distal side of the implant in the intervention group when compared with that of the control group but to a less extent (Table 5, Figure 5).

### 3.4. Predicting High Implant Stability for FD Measurements

**Figure 6:**
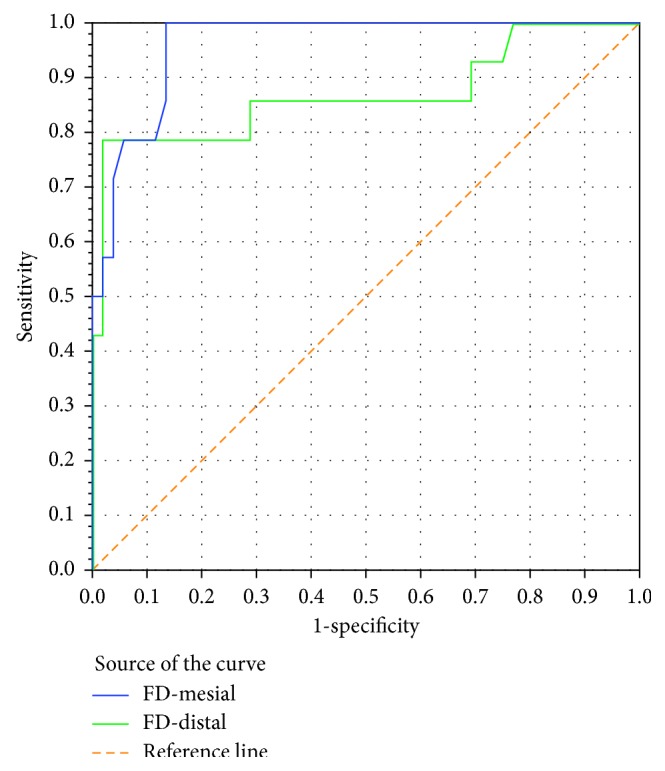
ROC curve showing the trade-off between sensitivity (rate of true positive results) and 1-specificity (rate of false positive results) for FD-mesial and FD-distal sides when used as a test to predict high implant stability (RF ≥ 70) in a sample of 66 paired measurements.

As shown in Figure 6 and Table 6, both FD-mesial and FD-distal sides measurements serve as very good-to-excellent tests with high validity (ROC area exceeding 0.8) for predicting high implant stability (ISQ ≥ 70). The mesial side measurements were consistently better than the distal side in this context.

As shown in Table 7, the optimum cutoff value for the FD-mesial side (associated with highest overall accuracy) that predicts a highly stable implant (ISQ ≥ 70) is ≥1.505. At this optimum cutoff value, the mesial side FD value is associated with a perfect sensitivity (100%) and fairly high specificity (86.5%). The positive predictive value of a positive test result (predicting a real highly stable implant) was calculated at a pretest probability of 50% (equal odds for having high stability versus not having based on chance alone), while that of the negative predictive value of the test (excluding the possibility of having high implant stability) was set at 10% (needing a clinical awareness of high probability of the implant being of low stability).

Having a positive test result for FD-mesial at the optimum cutoff value (FD-mesial measurement of 1.505 or higher) will establish high implant stability with 88.1% confidence level, while testing negative would exclude high implant stability with 100% confidence level. The highest specificity (100%) cutoff value for FD-mesial is ≥1.667. Testing positive at this diagnostic cutoff value (FD-mesial measurement of 1.667 or higher) will establish the diagnosis of high implant stability with 100% confidence, (Table 5).

**Figure 7:**
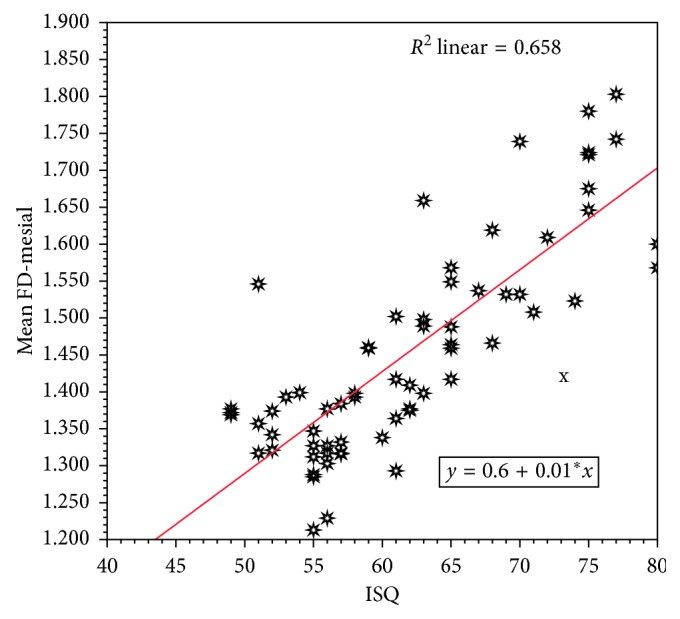
Scatter diagram with fitted regression line describing the linear relation correlation between RF- and FD-mesial side.

As shown in Table 8 and Figure 7, for each one-unit increase in FD-mesial side, the RF value is expected to increase by 47.7 units (for each 0.01-unit increase in measured FD, the RF value is expected to increase by around 0.5). The model is statistically significant and able to explain 66% of observed variation in RF values based on FD-mesial measurements. There was a strong and statistically significant linear correlation between RF values and FD-mesial (*r*=0.81).

**Figure 8:**
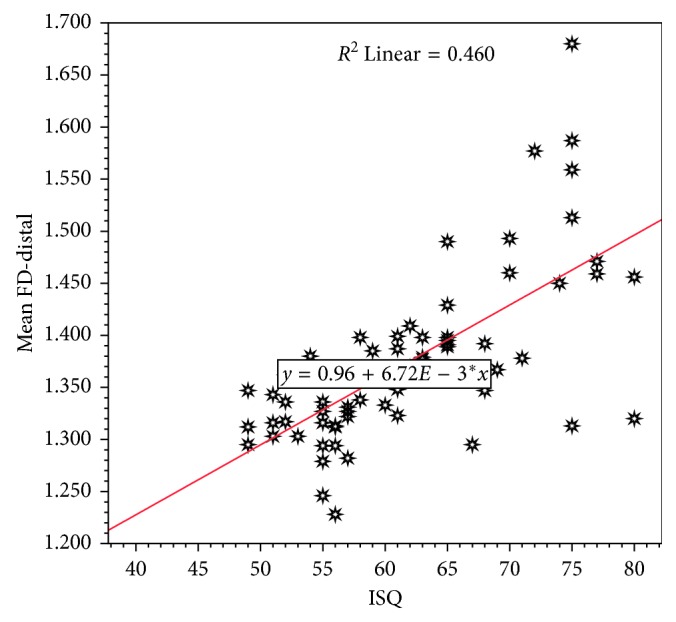
Scatter diagram with fitted regression line describing the linear relation correlation between RF- and FD-distal side.

As seen in Table 9 and Figure 8, for each one-unit increase in the FD-distal side, the RF value is expected to increase by 68.5 units. The model is statistically significant and able to explain 46% of observed variation in RF values based on FD-distal measurements. There was a statistically significant linear correlation between ISQ and FD-distal (*r*=0.678), but not as strong as that found in the mesial side.

## 4. Discussion

Clinically, the implant stability may be assessed either by recording of periotest value, insertion of torque wrench value, or by the use of the resonance frequency analysis. The RF analysis provides valuable clinical objective data of implant stability. It also detects substantial increase or decrease in stability of the implant, giving a clear ability to measure implant-bone contact and makes clinical comparisons during clinical follow-up. In the present study, all the implant fixtures used had the same surface treatment, implant-abutment interface, and thread characteristics. Regarding the ISQ values obtained from the RF analysis, the effect size after six months was much stronger for the LIPUS-treated intervention group (Cohen's *d* = 7.4) the compared to the control group (Cohen's *d* = 2.25). These findings were in agreement with that of Nedir et al. [25]. However, scarce evidence has been provided so far on Osstell ISQ's reliability [26]. The RF method is influenced by some factors including implant length, implant diameter, implant geometry, implant surface characteristic, and placement position, as well as bone quality and bone quantity [27]. Implant stability quotient (ISQ) is a scale developed by Osstell for implant stability. It converts the resonance frequency values ranging from 3,500 to 8,500 Hz into an ISQ of 0 to 100. A high value indicates greater stability, while a low value indicates instability. Values greater than 65 are recommended as successful implant stability. Even though Osstell is clinically used, there are not much convincing data on the relation between bone-implant interface and ISQ values [28, 29]. RF devices still have some uncertainties because of the observer subjectivity. There has been a search for quantitative methods to control the subjectivity variation in interpretation.

The reliability of fractal dimension calculations from the radiographs was assessed in several studies [15, 30]. Calculation of FD has become a popular method to characterize image textures. In dental radiology, FD calculation can be used to quantify trabecular bone structure for detection of bone changes associated with periodontitis [17], periapical pathology [15], systemic diseases [24, 31], and dental implants [19, 32]. We used standardized digital intraoral radiographs to assess the FD on two implant's protocols. Tolga et al. [32] suggested that fractal analysis could be a useful method for understanding the healing process around implants and implant stability quotient. Onem et al. [33] measured the FD around implants using the box-counting method from the digital panoramic images, and they found that FD values of implant-bearing bone following initial healing was lower than the FD values in the alveolar bone surrounding the contralateral premolar teeth, but the difference was not significant. In the present study, the fractal dimension after 6 months of implant placement had increased significantly (*P* < 0.01) in both groups, suggesting an increase in the amount of bony microstructure around the implant [20]. This increase suggests complete bone healing around implants. Veltri et al. [19] used animal models to investigate the correlations between FA results and insertion torque and RF. A significant correlation was found with final insertion torques, but not with resonance frequencies. Their results also defined a value of 1.83 as the break point of soft bone quality. Veltri et al. [34] measured FD from mesial and distal aspects of implants using the box-counting method on intraoral radiographs, and the mean reported was 1.47 which is close to what was reported in the present study after 6 months of implant placement (1.39–1.45). Lee et al. [18] observed a strong relationship between primary implant stability and FD using digitized panoramic films and the tile-counting method.

In the present study, there was a statistically significant linear correlation between the ISQ values from the RF and the FD values on both sides of the implant with the mesial side of the intervention group being higher than that on the distal side. This could be due to the large surface area of the LIPUS device used in the present study which provides stronger stimulation on the site closer to the probe (the mesial site of the implant); therefore, denser bone formation was expected on this site on a long run. Nevertheless, the FD values on the mesial and distal sides of the implant remains as very good-to-excellent tests with high validity (ROC area exceeding 0.8) for predicting high implant stability (ISQ > 70%). Our results were in agreement with that of Ilhan et al. [35] Lee et al. [18] also suggested that the FD acquired from panoramic radiographs may be a useful predictor of the initial dental implants stability. Therefore, the use of the FD method as a cost-effective one to assess trabecular bone changes around dental implants during follow-up periods is one of the recommendations of this study. No previous studies have been conducted to calculate the optimum cutoff value of FD to predict high implant stability (ISQ > 70%). Using the area under the ROC curve, showed that the optimum cutoff FD value of 1.667 from the mesial side of the implant will establish high implant stability with 100% confidence. Furthermore, for every one-unit increase in the FD-mesial side, the ISQ value from the RF is expected to increase by 47.7 units. Because no other studies have predicted the implant stability from combining the qualitative and quantitative bone at bone-implant interface from digital intraoral radiographs, it is not possible to compare these results with other studies. Onem et al. [33] have used digital panoramic radiographs to quantify the structural changes of mandibular alveolar bone around the dental implants during initial healing. They found that satisfactory bone healing after the implant placement may be monitored by calculating the FD. Lee et al. [18] suggested a unified method to calculate the FD, like proper selection of the area and the size of ROI, methods of FD counting (tile-or box-counting methods), and the type of radiographic image used for the analysis. Although these results may be regarded as preliminary, both ISQ from RF and FD may be reliably recommended for quantitative evaluation of primary and secondary implant stability using digital intraoral radiographic images throughout implant healing and loading periods. Application of the standardized procedures and comparison of the results of these methods with other measures define the bone quality like DEXA, and the value of FD would be more precisely assessed.

## 5. Conclusion

The fractal dimension analysis as a noninvasive cost-effective method could be helpful in assessing bone trabecular patterns around the implants in different clinical situations. Intraoral digital radiographs could be used to monitor the healing process around the implants. Although these results may be regarded as preliminary, implants with low FD values may indicate a decrease in stability, and this should alert the practitioner to pay more attention through more rigorous follow-up schedules and take further precautions.

## Figures and Tables

**Table 1:** Age and gender comparison between the intervention and control groups.

	Study group				*P*
	Control		Intervention		
	*N*	%	*N*	%	
Age group (years)					0.67 (NS)
21–30	4	36.4	5	45.5	
31–40	7	63.6	6	54.5	
Total	11	100.0	11	100.0	
Gender					1 (NS)
Female	7	63.6	7	63.6	
Male	4	36.4	4	36.4	
Total	11	100.0	11	100.0	

**Table 2:** The means and standard deviations of RF and FD in the intervention and control groups.

	RF Values (mean ± SD)			FD values (mean ± SD)			FD values (mean ± SD)		
	0 month	3 months	6 months	0 month	3 months	6 months	0 month	3 months	6 months
				Mesial side			Distal side		
Control group	55.3 ± 1.90	59.45 ± 4.50	62.09 ± 3.83	1.18 ± 0.19	1.35 ± 0.08	1.44 ± 0.06	1.06 ± 0.16	1.24 ± 0.07	1.36 ± 0.04
Intervention group	53.2 ± 2.99	65.91 ± 6.30	75.45 ± 3.01	1.42 ± 0.10	1.56 ± 0.06	1.68 ± 0.09	1.24 ± 0.07	1.35 ± 0.06	1.49 ± 0.11

RF: resonance frequency; FD: fractal dimension.

**Table 3:** An intervention-control group comparison in mean change in the RF after three and six months of surgery compared to immediate postoperative measurements.

	At baseline	After 3 months	Changes after 3 months compared to immediate	Cohen's *d*	*P* (paired *t*-test)	After 6 months	Changes after 6 months compared to immediate	Cohen's *d*	*P* (paired *t*-test)
*Control*									
Range	(52 to 58)	(49 to 65)	(−6 to 11)			(54 to 68)	(−1 to 13)		
Mean	55.3	59.5	4.2	1.21	0.009	62.1	6.8	2.25	<0.001
SD	1.9	4.5	4.3			3.8	3.9		
SE	0.57	1.36	1.30			1.16	1.19		
*N*	11	11	11			11	11		
*Intervention*									
Range	(49 to 57)	(51 to 75)	(2 to 19)			(70 to 80)	(15 to 28)		
Mean	53.2	65.9	12.7	2.58	<0.001	75.5	22.3	7.42	<0.001
SD	3.0	6.3	4.9			3.0	3.5		
SE	0.90	1.90	1.48			0.91	1.05		
*N*	11	11	11			11	11		
*P* (independent samples *t*-test)	0.06 (NS)	0.012	<0.001			<0.001	<0.001		

**Table 4:** An intervention-control group comparison in mean change in the FD-mesial side after three and six months of surgery compared to immediate postoperative measurements.

	At baseline	After 3 months	Changes after 3 months compared to immediate	Cohen's *d*	*P* (paired *t*-test)	After 6 months	Changes after 6 months composed to immediate	Cohen's *d*	*P* (paired *t*-test)
*Control*									
Range	(1.229 to 1.393)	(1.293 to 1.498)	(0.018 to 0.124)			(1.377 to 1.568)	(0.06 to 0.26)		
Mean	1.319	1.376	0.056	1.13	<0.001	1.452	0.132	2.64	<0.001
SD	0.044	0.056	0.032			0.052	0.059		
SE	0.013	0.017	0.010			0.016	0.018		
*N*	11	11	11			11	11		
*Intervention*									
Range	(1.213 to 1.393)	(1.488 to 1.659)	(0.145 to 0.319)			(1.523 to 1.803)	(0.181 to 0.462)		
Mean	1.338	1.556	0.218	4.35	<0.001	1.680	0.342	4.88	<0.001
SD	0.050	0.058	0.061			0.092	0.097		
SE	0.015	0.018	0.018			0.028	0.029		
*N*	11	11	11			11	11		
*P* (independent samples *t*-test)	0.36 (NS)	<0.001	<0.001			<0.001	<0.001		

**Table 5:** An intervention-control group comparison in mean change in the FD-distal side after three and six months of surgery compared to immediate postoperative measurements.

	At baseline	After 3 months	Changes after 3 months compared to immediate	Cohen's *d*	*P* (paired *t*-test)	After 6 months	Changes after 6 months compared to immediate	Cohen's *d*	*P* (paired *t*-test)
*Control*									
Range	(1.228 to 1.362)	(1.312 to 1.398)	(0.015 to 0.12)			(1.363 to 1.49)	(0.036 to 0.152)		
Mean	1.308	1.354	0.047	1.55	<0.001	1.398	0.090	2.25	<0.001
SD	0.036	0.031	0.031			0.036	0.038		
SE	0.011	0.009	0.009			0.011	0.011		
*N*	11	11	11			11	11		
*Intervention*									
Range	(1.246 to 1.347)	(1.295 to 1.493)	(0.008 to 0.15)			(1.32 to 1.68)	(0.074 to 0.386)		
Mean	1.315	1.370	0.055	1.38	<0.001	1.503	0.188	2.68	<0.001
SD	0.029	0.056	0.037			0.095	0.087		
SE	0.009	0.017	0.011			0.029	0.026		
*N*	11	11	11			11	11		
*P* (independent samples *t*-test)	0.61 (NS)	0.42 (NS)	0.56 (NS)			0.003	0.003		

**Table 6:** Area under ROC curve for FD-mesial and FD-distal sides when used as a test to predict high implant stability (ISQ ≥ 70).

	AUROC	*P*
FD-mesial	0.962	<0.001
FD-distal	0.869	<0.001

**Table 7:** Validity parameters for FD-mesial and FD-distal sides when used as a test to predict high implant stability (ISQ ≥ 70).

Positive if ≥ cutoff value	*k*	Specificity	Accuracy	PPV at pretest probability = 50%	NPV at pretest probability = 10%
*FD-mesial*					
1.505 (highest sensitivity and optimum cutoff value)	100.0	86.5	96.2	88.1	100.0
1.516	92.9	86.5	91.1	87.3	99.1
1.653	50.0	98.1	63.7	96.3	94.6
1.667 (highest specificity)	50.0	100.0	64.3	100.0	94.7
*FD-distal*					
1.313 (highest sensitivity)	100.0	23.1	78.0	56.5	100.0
1.315	92.9	25.0	73.5	55.3	96.9
1.419	78.6	96.2	83.6	95.3	97.6
1.440 (optimum cutoff value)	78.6	98.1	84.1	97.6	97.6
1.481	42.9	98.1	58.6	95.7	93.9
1.492 (highest specificity)	42.9	100.0	59.2	100.0	94.0

**Table 8:** Simple linear regression model for FD-mesial side as a predictor variable and RF values as the dependent (outcome) variable.

	Regression coefficient	*P*
(Constant)	−7.5	0.24 (NS)
FD-mesial	47.7	<0.001

*R* ^2^=0.66, *P*  (model) < 0.001, *r*=0.811, and *P* < 0.001.

**Table 9:** Simple linear regression model for the FD-distal side as a predictor variable and RF as the dependent (outcome) variable.

	Regression coefficient	*P*
(Constant)	−32.3	0.014
FD-distal	68.5	<0.001

*R* ^2^=0.46, *P*  (*Model*) < 0.001, *r*=0.678, *P* < 0.001.
